# Ovaries vs. Testicles

**Published:** 1888-12

**Authors:** Edward Borck

**Affiliations:** St. Louis, Mo.


### OVARIES VS. TESTICLES.

BY EDWARD BORCK, A. M., M. D., ST. LOUIS, MO.

In a paper read before the Missouri State Medical Association, April 18th, 1888, the author discussed the neglect of the testicles as compared with the ovaries, advised the formation of a new specialism called testicology, reviled the great amount of fuss made over a little trouble in the female generative organs and said the blame in non-productive unions was far too frequently laid at the door of the wife instead of the husband. He was decidedly opposed to every new graduate rushing off to Europe and studying some special line for a few months and then setting up as a specialist. He reported the following interesting cases:

CASE I.—Mrs. ———, a healthy, handsome young woman, her husband apparently well, but after four years trial still they have no children. The family physician exhausted his ingenuity and his stock of prescriptions but all in vain; no baby came to bless this household with its smiles. Now the case is referred to the gynecologist, surely he can find out the trouble and give relief. He discovered at once a little tilting of the womb, only the one-sixtieth part of an inch it is true, but there was also some ulceration of the cervix. Pessaries are introduced, iodine applied locally to the back of the womb for months. Medicines were purchased by the small basket full. Every time the local applications were made to the womb Madame was assured by the gynecologist that she was getting better, and it would not be long to wait for the baby. (Special skill.) But Madame herself did not agree with him. In her opinion she was getting worse all the time. At last her mind wandered, she became emaciated and the case was taken from the gynecologist and went into the hands of a first-class neurologist. He promptly diagnosed the whole trouble. It was not the womb at fault: it was the nervous system. He was right, sure enough, for by this time it was the nervous system. Now came all the nerve tonics, sea bathing, travel, etc.; everything was done that could be thought of, even artificial fecundation was done but it was all to no purpose. The poor woman was now almost insane and still there was no baby. Having failed of success the neurologist goes for his predecessor, the gynecologist, and shows his lack of skill and general ignorance in his profession.

Last of all the case was placed in my hands. Every practitioner is called upon to explain the cause of barrenness and to treat and cure, but very often I am sorry to say he is called upon to prevent pregnancy or to cure (kill) it when present; the latter the most disgusting thing in female practice. After examining this patient carefully I found the gynecologist was right in his diagnosis, the womb was one-sixtieth of an inch out of the way and there was a little irritation of the uterus.

But was this the cause of the sterility? Not by a long way. Many a womb deviates a little from the imaginary natural line and they become pregnant all the same and more than once. We find plenty of men with crooked noses but they smell straight. Why cannot a womb with a little crook in it become impregnated? But to return to our patient. She exclaimed: "Oh Doctor, if I could only become pregnant and have a boy baby I would get well and my husband would be satisfied." I consoled her with the hope that she would get well in time and dismissed her telling her to send the husband to me. Upon inquiry of him, he stated that in his opinion the act of coitus was not perfect, "too soon or often nothing came at all," as he expressed it. Found that he was disposed to fatty generation of the heart; had fatty deposits in the scrotum and testicles and fatty degeneration of the spermatozoa. I advised separation for a time from his wife. To shorten the story, upon the return of the husband from abroad after a six month's separation, both were well; she became enciente shortly afterwards, happiness once more prevailed and a boy baby was born to the exultant pair.

CASE 2.—The wife was treated for sterility without any benefit. I found the fault with the husband; his penis was of very small size; he informed me it was growing smaller all the time. I found a large cyst of the left testicle which prevented the proper introduction of the penis into the vagina. None of of the numerous male doctors who had treated the wife for barrenness had ever inquired into the condition of the husband's genital organs. To be brief, I removed the cyst, the parts became of natural size, and in due time his wife presented him with not only one, but twin babies.

CASE 3.—The wife unsuccessfully treated for sterility. The fault was with the husband again, and through he was the son of a physician and had congenital phymosis, he never did or never could enjoy the act of coitus. Circumcision relieved the man of his ailment, and the result was a child.

CASE 4.—Reflex nervous affections produced by disease of the testicles.

A merchant about 40 years old, married, with a happy family about him, became sleepless, languid, wandering, hypochondriac, ready to commit suicide. Cause of trouble attributed to business by a skillful neurologist. He was sent to a private retreat, but upon return to family and business the old story again. Then he consulted me, and his wife informed me of all the previous circumstances. They insisted upon having my advice, so I began to examine him with surgical eyes. All my questions as to injuries received upon the head, spine, etc., were answered negatively. Then I had the gentleman step into the next room and remove all clothing, which he did with great reluctance. The result was the production of a very large hypertrophied left testicle and he told me that years ago he had epididymitis. Here at last was brought out the whole cause of the man's brain trouble in a bag. I asked him if he had told his previous doctors of this complaint. "No sir; I did not like to." After a great deal of hesitation on his part, my advice of the removal of the diseased testicle was followed. All nervous trouble soon ceased; peace, tranquility and prosperity again reigned; a large new store was erected and, best of all, he soon had an addition made to his family.

I could give more and similar cases; these are sufficient for illustration.

In regard to neuralgic pains of the ovaries, viz: the testicles, both sexes suffer from the pains. When all known means for the relief of a woman's pain have been tried and failed and the removal of the offending ovary or ovaries has been advised, she will readily and cheerfully consent. Under the same conditions the man is not willing to give up one testicle, far less both, he prefers to suffer most excruciating pain until at last he lands in the insane asylum. Neither is the removal of such a painful organ advised by the physician, or if proposed it is done reluctantly. Why? Can any one give a definite answer.

The removal of a testicle or any operation on their appendages is not nearly so dangerous as the removal of one of the ovaries or any other operation upon the female reproductive organs.

The operation for variocele, which I now prefer, is to cut down upon it under strict antiseptic precautions, ligate the veins above and below, excise the distended part and close the wound.

I hope that someone who has had ten or fifteen years large general practice will give the disease of the testicles and operations thereon his particular attention and study and adopt it as a specialty. —*American Lancet.*

